# Identification of stably expressed microRNAs in plasma from high-grade serous ovarian carcinoma and benign tumor patients

**DOI:** 10.1007/s11033-023-08795-6

**Published:** 2023-11-07

**Authors:** Patrick H.D. Petersen, Joanna Lopacinska-Jørgensen, Claus K. Høgdall, Estrid V. Høgdall

**Affiliations:** 1 Department of Pathology, Herlev Hospital, University of Copenhagen, Borgmester Ib Juuls Vej 25, Herlev, 2730 Denmark; 2 Department of Gynecology, The Juliane Marie Centre, Rigshospitalet, University of Copenhagen, Copenhagen, 2100 Denmark

**Keywords:** microRNA (miRNA), Epithelial ovarian cancer, Endogenous controls, Normalization, RT-qPCR

## Abstract

**Background:**

Ovarian cancer is a lethal gynecological cancer and no reliable minimally invasive early diagnosis tools exist. High grade serous ovarian carcinoma (HGSOC) is often diagnosed at advanced stages, resulting in poorer outcome than those diagnosed in early stage. Circulating microRNAs have been investigated for their biomarker potential. However, due to lack of standardization methods for microRNA detection, there is no consensus, which microRNAs should be used as stable endogenous controls. We aimed to identify microRNAs that are stably expressed in plasma of HGSOC and benign ovarian tumor patients.

**Methods and results:**

We isolated RNA from plasma samples of 60 HGSOC and 48 benign patients. RT-qPCR was accomplished with a custom panel covering 40 microRNAs and 8 controls. Stability analysis was performed using five algorithms: Normfinder, geNorm, Delta-Ct, BestKeeper and RefFinder using an R-package; RefSeeker developed by our study group [1]. Among 41 analyzed RNAs, 13 were present in all samples and eligible for stability analysis. Differences between stability rankings were observed across algorithms. In HGSOC samples, hsa-miR-126-3p and hsa-miR-23a-3p were identified as the two most stable miRNAs. In benign samples, hsa-miR-191-5p and hsa-miR-27a-3p were most stable. In the combined HGSOC and benign group, hsa-miR-23a-3p and hsa-miR-27a-3p were identified by both the RefFinder and Normfinder analysis as the most stable miRNAs.

**Conclusions:**

Consensus regarding normalization approaches in microRNA studies is needed. The choice of endogenous microRNAs used for normalization depends on the histological content of the cohort. Furthermore, normalization also depends on the algorithms used for stability analysis.

**Supplementary Information:**

The online version contains supplementary material available at 10.1007/s11033-023-08795-6.

## Introduction

Ovarian cancer (OC) is the most lethal gynecological cancer with an estimated 313,959 new cases and 207,252 deaths worldwide in 2020 [2]. OC is subdivided into four main stages by the International Federation of Gynecology and Obstetrics (FIGO). Epithelial OC (EOC) which accounts for about 90–95% of OCs consists of four main histological subtypes: 75% Serous Carcinoma (SC) of which 70% is High grade (HGSOC) and 5% is Low Grade (LGSC), 10% are Clear Cell Carcinomas, 10% are Endometroid Carcinomas, and about 5% are Mucinous Carcinomas. Most HGSOCs are diagnosed in the late stages (FIGO III and IV) due to subtle symptoms of disease [3]. This is the most predominant and aggressive type of OC and due to high incidence and low survival rates caused by late-stage discovery, HGSOC is the most lethal of the EOCs [4], accounting for 70–80% of death related to ovarian cancer [5]. These cancers are characterized by frequent DNA gains and losses and chromosomal instability causing gene breakage and loss of hetero- and homozygosity [5].

The late discovery of HGSOC has a significant negative impact on overall survival and hence, there is an urgent clinical need for diagnostic and prognostic biomarkers. MicroRNAs (miRNAs) are small, noncoding RNAs and due to their involvement in regulation of mRNA and protein expression have gained increasing attention as biomarkers for various diseases [6–8]. However, the implementation of miRNA biomarkers in a clinical setting has been progressing slowly, in part due to missing standardization of methods for measuring, detecting and normalizing miRNA expression [9].

An important part of this standardization also pertains to the preprocessing of the data. Melt curve analysis is performed to identify poorly amplified products, the existence of primer dimer or genomic contamination. Besides being quite time consuming, melt curve evaluation is often very subjective. Spike-in controls is used by many to monitor efficiency and quality of RNA extraction, cDNA synthesis and final amplification. Many claim to perform these analyses but do not report methodology or list exclusion criteria and in our experience, it is difficult to locate specific guidelines and general acceptable cut-off and threshold values for variation in spike-in controls or differentiating between an insignificant shoulder and a second peak in a melt curve.

Ideally, normalization should be performed using stable endogenous references of the same type of RNA that is being quantified [10, 11]. However, identification of suitable references for normalization is not a trivial exercise. There is no universal endogenous control suitable for every tissue type, as the expression of most miRNAs varies with cell type and condition [12, 13]. An example of this is U6 (RNU-6-1) that have commonly been utilized as an endogenous control for miRNA expression in OC [14, 15] even though it has been shown to be differentially expressed in cancers and plasma [16]. Instead, references should be validated on a per study basis and several algorithms, assessing stability from RT-qPCR data are freely available. Normfinder [17], and geNorm [18] seem to be the most used but also delta-Ct [19] and Bestkeeper [20] have been used extensively. Normfinder ranks the stability of target RNAs based on a weighted geometric mean of the inter- and intra-group variations. GeNorm calculates a stability value M based roughly on the standard deviation of the linearized pairwise ratios between each target for each sample. Delta-Ct uses candidate pairwise ΔCt values, estimating a mean standard deviation for each candidate. BestKeeper stability is based on the mean absolute deviation (MAD) of each candidate. These different approaches may lead to different ranking of candidate references and discrepancy between research groups, comparing results from several algorithms may increase confidence in the selected reference [21]. Various combinations of the four have been employed. In the case with RefFinder [22], all these four algorithms are taken into account when calculating a geometric mean of the candidate’s stability rankings from the different algorithms. Ultimately, to advance the discovery and clinical utility of miRNA expression as biomarkers, consensus regarding these methodologies need to be build. There are no published studies on identification of stably expressed endogenous miRNAs in OC. In this study we aimed to provide a starting framework for discussing these methodological deficiencies while investigating stability of selected miRNAs in plasma samples from HGSOC patients and patients with benign gynecological tumors.

## Materials and methods

### Study design

EDTA plasma samples were collected prior to primary surgery from 60 patients diagnosed with ovarian high grade serous carcinoma (HGSOC) and 48 patients with benign gynecological tumors relevant to our clinical setting. Samples were obtained through the Bio- and Genome Bank Denmark from two Danish projects: the Pelvic Mass study (2004–2014) and the GOVEC (Gynecological Ovarian Vulva Endometrial Cervix cancer) study (2015 – ongoing). Each patient has provided a written informed consent and the Danish National Committee for Research Ethics, Capital Region has approved the study (approval codes KF01-227/03 and KF01-143/04). During this study the Declaration of Helsinki guidelines was followed.

### miRNA extraction of plasma samples

RNA was extracted using the miRNeasy Serum/Plasma Kit (Qiagen, Copenhagen, Denmark, cat. no. 217,184) as previously described [21]. Briefly, 200 µl plasma was lysed using 1 ml QIAzol lysis reagent, followed by addition of 1 µl RNA isolation control spike-ins mix consisting of UniSp2, UniSp4 and UniSp5, each at a different concentration with 100-fold increments: UniSp2 > UniSp4 > UniSp5. Afterwards, the samples were purified according to the manufacturer’s recommendations. RNA was eluted in 14 µl RNase free water and stored at -80 °C until further use. A no sample control extraction was also performed. This sample was subjected to the same procedure, only no plasma was added to the lysis buffer.

### cDNA synthesis

cDNA synthesis was performed using miRCURY LNA RT Kit (Qiagen, Copenhagen, Denmark, cat. no. 339,340) following the manufacturer’s protocol. Briefly, for each 10 µl reaction, 2 µl 5X reaction buffer, 1 µl 10X miRCURY RT enzyme mix as well as 5.4 µl nuclease free water, 1.1 µl RNA and 0.5 µl RNA cDNA synthesis spike-in control mix containing UniSp6 and cel-miR-39-3p.

### RT-qPCR

For RT-qPCR, on blood-plasma samples we decided to use a custom miRCURY LNA miRNA PCR Panels (Qiagen, Copenhagen, Denmark) previously designed for investigation miRNA expression in tissue samples from ovarian cancer patients [23]. See table S1. The PCR panel contained 40 miRNAs selected for their reported stability or potential as biomarkers in OC. Also, U6 (RNU6-1) which has previously been used as a reference for normalization was included. Assays for the spike-ins controls UniSp2, UniSp4 and UniSp5 added during RNA extraction as well as cel-miR-39-3p and UniSp6 that were added during cDNA synthesis were also selected. Additionally, the interplate calibrator UniSp3 was included to correct for inter plate variations as well as a blank spot to control for possible contaminations. A list of analyzed miRNAs can be found in Table 1 and further details regarding selection of these can be found in supplementary Table S1.

**Table 1:** List of analyzed miRNAs and controls

miRNA	MiRCury_Assay_cat._no.	microRNA target sequence	Assay type
UniSp6	YP00203954		Spike
UniSp2	YP00203950		Spike
UniSp4	YP00203953		Spike
UniSp5	YP00203955		Spike
cel-miR-39-3p	YP00203952		Spike
UniSp3	YP02119288		Spike
U6 snRNA (V2)	YP02119464		GOI
hsa-miR-101-3p	YP00204786	UACAGUACUGUGAUAACUGAA	GOI
hsa-miR-103a-3p	YP00204063	AGCAGCAUUGUACAGGGCUAUGA	GOI
hsa-miR-106b-3p	YP00204020	CCGCACUGUGGGUACUUGCUGC	GOI
hsa-miR-1183	YP00204176	CACUGUAGGUGAUGGUGAGAGUGGGCA	GOI
hsa-miR-1234-3p	YP00206017	UCGGCCUGACCACCCACCCCAC	GOI
hsa-miR-126-3p	YP00204227	UCGUACCGUGAGUAAUAAUGCG	GOI
hsa-miR-1301-3p	YP02111482	UUGCAGCUGCCUGGGAGUGACUUC	GOI
hsa-miR-130a-3p	YP00204658	CAGUGCAAUGUUAAAAGGGCAU	GOI
hsa-miR-135a-3p	YP00204022	UAUAGGGAUUGGAGCCGUGGCG	GOI
hsa-miR-139-3p	YP00205661	UGGAGACGCGGCCCUGUUGGAGU	GOI
hsa-miR-141-3p	YP00204504	UAACACUGUCUGGUAAAGAUGG	GOI
hsa-miR-143-3p	YP00205992	UGAGAUGAAGCACUGUAGCUC	GOI
hsa-miR-146b-5p	YP02119310	UGAGAACUGAAUUCCAUAGGCUG	GOI
hsa-miR-149-3p	YP00204093	AGGGAGGGACGGGGGCUGUGC	GOI
hsa-miR-191-5p	YP00204306	CAACGGAAUCCCAAAAGCAGCUG	GOI
hsa-miR-193a-5p	YP00204665	UGGGUCUUUGCGGGCGAGAUGA	GOI
hsa-miR-195-5p	YP00205869	UAGCAGCACAGAAAUAUUGGC	GOI
hsa-miR-199a-3p	YP00204536	ACAGUAGUCUGCACAUUGGUUA	GOI
hsa-miR-199a-5p	YP00204494	CCCAGUGUUCAGACUACCUGUUC	GOI
hsa-miR-200b-3p	YP00206071	UAAUACUGCCUGGUAAUGAUGA	GOI
hsa-miR-200c-3p	YP00204482	UAAUACUGCCGGGUAAUGAUGGA	GOI
hsa-miR-205-5p	YP00204487	UCCUUCAUUCCACCGGAGUCUG	GOI
hsa-miR-21-5p	YP00204230	UAGCUUAUCAGACUGAUGUUGA	GOI
hsa-miR-221-3p	YP00204532	AGCUACAUUGUCUGCUGGGUUUC	GOI
hsa-miR-223-3p	YP00205986	UGUCAGUUUGUCAAAUACCCCA	GOI
hsa-miR-23a-3p	YP00204772	AUCACAUUGCCAGGGAUUUCC	GOI
hsa-miR-23a-5p	YP00205631	GGGGUUCCUGGGGAUGGGAUUU	GOI
hsa-miR-24-2-5p	YP00204187	UGCCUACUGAGCUGAAACACAG	GOI
hsa-miR-24-3p	YP00204260	UGGCUCAGUUCAGCAGGAACAG	GOI
hsa-miR-27a-3p	YP00206038	UUCACAGUGGCUAAGUUCCGC	GOI
hsa-miR-27a-5p	YP00206021	AGGGCUUAGCUGCUUGUGAGCA	GOI
hsa-miR-302d-3p	YP00204311	UAAGUGCUUCCAUGUUUGAGUGU	GOI
hsa-miR-34a-5p	YP00204486	UGGCAGUGUCUUAGCUGGUUGU	GOI
hsa-miR-455-3p	YP00204035	GCAGUCCAUGGGCAUAUACAC	GOI
hsa-miR-486-5p	YP00204001	UCCUGUACUGAGCUGCCCCGAG	GOI
hsa-miR-506-3p	YP00204539	UAAGGCACCCUUCUGAGUAGA	GOI
hsa-miR-595	YP00204070	GAAGUGUGCCGUGGUGUGUCU	GOI
hsa-miR-665	YP00204710	ACCAGGAGGCUGAGGCCCCU	GOI
hsa-miR-802	YP00205980	CAGUAACAAAGAUUCAUCCUUGU	GOI
hsa-miR-92b-5p	YP00204415	AGGGACGGGACGCGGUGCAGUG	GOI

RT-qPCR reactions were performed as previously described [21] using the custom miRCURY LNA miRNA PCR Panels, in a 384-well plate format (Qiagen, Copenhagen, Denmark) and a LightCycler 480 384-well Block (Roche, Hvidovre, Denmark).

For each sample, a solution containing 264 µl 2X miRCURY SYBR Green master mix, 257 µl nuclease free water and 7 µl cDNA was prepared. Eight samples were prepared for each 384-well plate containing primers and polymerase enzymes and 10 µl reaction mix was aliquoted to each well. The PCR plates were then sealed, centrifuged for 1 min at 1500 x g, and subjected to real-time PCR amplification in a Roche LightCycler 480 according to the protocol, including 2 min heat activation at 95 °C, 45 amplification cycles of 95 °C for 10 s and 56 °C for 60 s ending with a melt curve analysis.

Crossing points (Cps) of the amplification curves were calculated by the LightCycler®480 software version 1.5 (Roche) using absolute quantification analysis/2nd derivative maximum method with high confidence setting. Melting temperature analysis (Tm calling) and calculating melting curve peaks were performed through the LightCycler®480 software. Cp and Tm tables as well as raw melt data were exported as txt-files for melt curve and data analysis.

### Data analysis

**Fig. 1:**
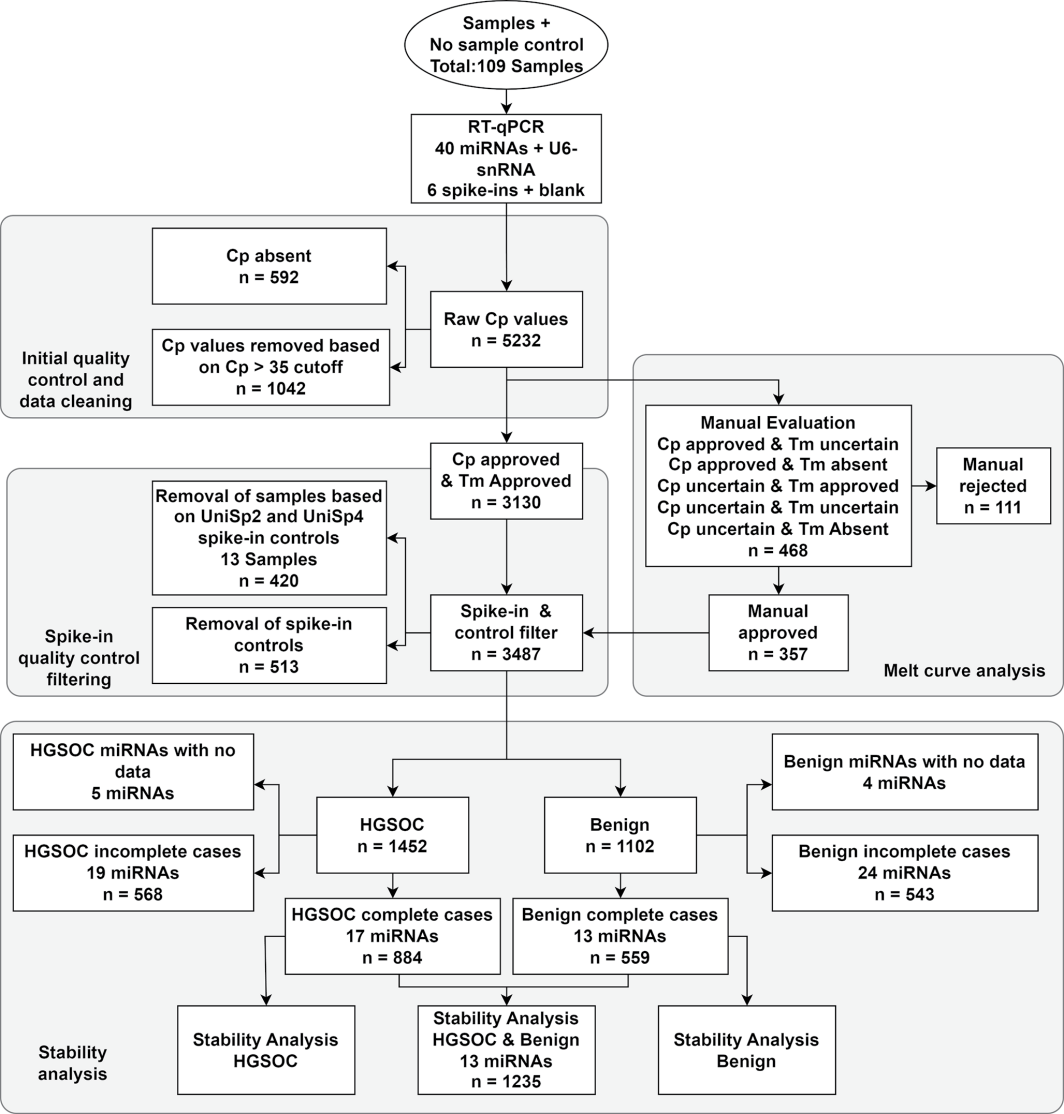
Workflow diagram explaining the quality control filtering process. Value in the first bubble represents number of samples (109 samples). All n values represent the number of Cp value data points arriving at the node. Note that when combining complete cases from the HGSOC and Benign group for stability analysis, only miRNAs that are in common in the two datasets are merged, meaning that, four miRNAs and 220 Cp values were removed from the HGSOC group

All data analyses were performed using R Statistical Software (version 4.1.1; R Foundation for Statistical Computing, Vienna, Austria) and R-studio IDE (version 1.4.1717, RStudio, Boston, United states, rstudio.com) and [24]. An overview of the analysis workflow can be seen in Fig. 1.

### Melt curve analysis

Melt curve analyses were performed to prevent using data from poorly amplified products caused by primer dimer, non-specific targets, or genomic contamination. Prior to analysis, an adjusted Cp value was calculated based on the inter plate calibrator; UniSp3 according to manufacturer’s instructions. A Cp cutoff value of 35 was chosen and all Cp or adjusted Cp values above this threshold were set to not available (NA). Four of the 41 analyzed RNAs were removed due to all Cp values being above this cut-off value.

The Cp table, the Tm table, and the melt curve data provided by the LightCycler®480 software was used to evaluate initial sample quality. Using the color and status column present in the exported Cp data, each reaction was characterized as either; approved, uncertain, absent, or late. For the Tm calling data, three categories were identified: approved, inconclusive, or absent. In this way, each reaction was categorized according to supplementary Table S2. Manual melt curve assessment was performed on amplifications where a Cp value was reported, but not auto-approved (approved by both the Cp and Tm calling produced by the LightCycler®480 software) (supplementary Table S2, grey shaded cells).

The manual evaluation was performed by comparing the melt curve for the reaction in question with all auto-approved melt curves from the same miRNA. In general, reactions were rejected manually if no clear peak was seen, a peak was seen but did not reach a threshold of 0.5 fluorescent units, a melt curve contained two peaks and one peak was at least half the size of the other or if more than two peaks were seen. A total of 468 melt curves were analyzed manually out of which 357 datapoints were approved, the rest were rejected and removed from the dataset Fig. 1.

### Spike-in analysis

To control for variation in extraction efficiencies, three spike-in RNAs (UniSp4, UniSp4 and UniSp5) were added to the lysis buffer during RNA extraction. Samples were split in two groups: HGSOC and benign. Based on these groups, samples were removed if their Cp value for UniSp2 was an outlier as defined by the ± 1.5xIQR rule [25] or if the difference in Cp values of UniSp2 and UniSp4 were not between five and eight cycles. UniSp5 was not considered since the Cp values for this spike-in were above Cp = 35 cut-off. Hemolysis was checked on 20 random samples using the miRCURY QC Panel according to the protocol and as described in [21, 26]. No hemolysis was observed and was therefore considered negligible.

A summary of the spike-in analysis can be seen in Supplementary Table S3, and an overview of the workflow can be seen in Fig. 1. No HGSOC sample was identified as a UniSp2 outlier, six samples that had a difference in UniSp2 and UniSp4 Cp values of less than five. Two samples had a difference in UniSp2 and UniSp4 Cp values of more than eight. In total, eight samples were excluded from the HGSOC group resulting in the removal of one miRNA and a total of 52 remaining. Of the benign samples, five had a difference in Cp values of UniSp2 and UniSp4 of less than five, one of which was also identified as a UniSp2 outlier. No samples had a difference in UniSp2 and UniSp4 Cp values of more than eight. In total, five samples were excluded from further analyses, leaving 43 samples for further analyses.

### Stability analysis and normalization

To investigate the stabilities of miRNAs, we developed the R package RefSeeker [1] based on the web tool RefFinder [22] found at https://www.heartcure.com.au/reffinder/. RefFinder utilizes four widely used algorithms for stability analysis; BestKeeper [20], Normfinder [17], GeNorm [18] and the comparative delta-Ct method [19]. Using the ranking of stability values from the four other algorithms RefFinder calculates a geometric mean, representing an overall stability ranking value for each miRNA. These algorithms do not perform equally well on all datasets. The geNorm algorithm tends to favor highly correlated genes excluding potential stable genes with bad correlation to regulated genes [27]. Compared to geNorm, Normfinder has been described as less robust with smaller sample sizes [28] and the delta-Ct method was found to work better with heterogeneously correlated sets of genes [27]. The full BestKeeper analysis calculates two main measures of stability, one of which is the raw MAD that is incorporated into the RefFinder method. The MAD, however, is best suited for samples with a very fixed amount of input material [28].

The RefFinder rank was calculated separately for each of the HGSOC and benign groups as well as the HGSOC and benign combined on miRNAs that were detected in all samples. Stabilities of candidate pairs were also checked using the Normfinder analysis of expression data grouped in benign and HGSOC samples.

## Results

### Patients

Clinical and pathologic characteristics of the patients are summarized in Table 2. For the control group, patients with various benign gynecological tumors relevant in the clinical setting was selected. A group of 60 HGSOC patients were selected as test group. Of these, one patient was categorized as FIGO stage II, 38 as FIGO stage III and 21 as FIGO stage IV. Outcome of the patients was checked on 2 November 2021.

**Table 2:** Clinical and pathological characteristics of the patients

	HGSOC	Benign
	n = 60	n = 48
**Age at diagnosis, median (Range)**	65.33 (38–71)	52.13 (18–68)
**Stage**		
II	1	
III	38	
IV	21	
**Histology**		
High grade serous adenocarcinoma	60	
Serous Cystadenoma		6
Muscinous Cystadenoma		3
Endometriosis		2
Fibroma		22
Functional/simple/haemorrhagic/paratubal/dermoid ov. Cysts.		9
Other		6

### Identification of stable miRNA

After quality control of the data, 17 miRNAs presented complete cases in the HGSOC group, 19 miRNAs had missing values and five miRNAs were excluded since all Cp values were eliminated (Table 3). In the benign group, 13 miRNAs were present in all remaining samples, 24 miRNAs had missing data and four miRNAs were eliminated due to removal of all Cp values (Table 4). Remarkably, U6 were missing in 56% of the HGSOC samples and 77% of the benign samples and were never considered as a candidate reference RNA.

**Table 3:** Overview of miRNAs that after quality control were present in all remaining HGSOC samples (complete cases), had missing values (incomplete cases) and no Cp values (excluded)

Complete Cases			Incomplete Cases				Excluded (no cases)
Target	Mean	Sd	Target	mean	Sd	Missing	Target
hsa-miR-101-3p	27.72	1.79	hsa-miR-1301-3p	30.21	1.83	2%	hsa-miR-1183
hsa-miR-103a-3p	24.94	2.20	hsa-miR-34a-5p	32.11	1.78	2%	hsa-miR-302d-3p
hsa-miR-126-3p	25.12	2.00	hsa-miR-200c-3p	31.42	1.93	6%	hsa-miR-455-3p
hsa-miR-130a-3p	27.26	2.09	hsa-miR-106b-3p	31.41	1.57	8%	hsa-miR-595
hsa-miR-143-3p	30.39	1.94	hsa-miR-195-5p	32.23	1.45	8%	hsa-miR-802
hsa-miR-146b-5p	29.72	2.12	hsa-miR-205-5p	32.40	1.76	8%	
hsa-miR-191-5p	25.08	1.91	hsa-miR-24-2-5p	32.01	1.68	19%	
hsa-miR-193a-5p	31.54	1.15	hsa-miR-1234-3p	29.06	1.43	25%	
hsa-miR-199a-3p	26.34	1.96	hsa-miR-139-3p	32.37	1.44	25%	
hsa-miR-199a-5p	28.64	2.14	hsa-miR-141-3p	32.61	1.68	31%	
hsa-miR-21-5p	24.55	2.02	hsa-miR-200b-3p	33.15	1.44	31%	
hsa-miR-221-3p	25.26	2.04	U6_snRNA_(v2)	34.03	0.92	56%	
hsa-miR-223-3p	22.52	2.00	hsa-miR-23a-5p	33.81	0.79	58%	
hsa-miR-23a-3p	24.38	2.05	hsa-miR-27a-5p	33.80	0.84	73%	
hsa-miR-24-3p	24.46	1.98	hsa-miR-506-3p	34.36	0.65	75%	
hsa-miR-27a-3p	26.12	2.12	hsa-miR-665	32.58	2.56	92%	
hsa-miR-486-5p	24.17	1.48	hsa-miR-149-3p	34.91	0.11	96%	
			hsa-miR-92b-5p	34.25	0.40	96%	
			hsa-miR-135a-3p	34.59		98%	

**Table 4:** Overview of miRNAs that after quality control were present in all remaining benign samples (complete cases), had missing values (incomplete cases) and no Cp values (excluded)

Complete Cases			Incomplete Cases				Excluded (no cases)
Target	Mean	Sd	Target	mean	Sd	Missing	Target
hsa-miR-101-3p	28.80	1.74	hsa-miR-199a-5p	29.51	1.97	2%	hsa-miR-1183
hsa-miR-103a-3p	25.52	2.40	hsa-miR-1301-3p	30.59	1.93	5%	hsa-miR-302d-3p
hsa-miR-126-3p	25.88	2.16	hsa-miR-146b-5p	30.51	1.88	5%	hsa-miR-595
hsa-miR-130a-3p	28.34	2.04	hsa-miR-106b-3p	31.88	1.95	7%	hsa-miR-802
hsa-miR-191-5p	25.91	2.00	hsa-miR-143-3p	31.36	2.07	7%	
hsa-miR-199a-3p	27.17	2.11	hsa-miR-1234-3p	30.18	1.78	9%	
hsa-miR-21-5p	25.53	2.06	hsa-miR-193a-5p	32.94	1.47	12%	
hsa-miR-221-3p	26.13	2.33	hsa-miR-24-2-5p	32.68	1.78	14%	
hsa-miR-223-3p	23.48	1.98	hsa-miR-200c-3p	32.10	1.73	19%	
hsa-miR-23a-3p	25.20	2.24	hsa-miR-139-3p	32.86	1.37	30%	
hsa-miR-24-3p	25.36	2.31	hsa-miR-34a-5p	32.81	1.33	35%	
hsa-miR-27a-3p	27.18	2.11	hsa-miR-195-5p	32.72	1.34	40%	
hsa-miR-486-5p	25.47	1.94	hsa-miR-205-5p	33.64	1.32	51%	
			hsa-miR-141-3p	33.15	1.36	56%	
			hsa-miR-200b-3p	33.23	1.15	63%	
			hsa-miR-23a-5p	33.83	1.45	63%	
			U6_snRNA_(v2)	34.08	1.03	77%	
			hsa-miR-665	33.76	0.80	77%	
			hsa-miR-27a-5p	34.43	0.43	91%	
			hsa-miR-506-3p	34.11	0.78	91%	
			hsa-miR-455-3p	34.66	0.22	95%	
			hsa-miR-92b-5p	34.76	0.18	95%	
			hsa-miR-135a-3p	34.16		98%	
			hsa-miR-149-3p	34.80		98%	

Stability assessment was performed on the complete cases using the RefFinder method through the R package RefSeeker [1]. This method provides results from the delta-Ct, BestKeeper, Normfinder and geNorm algorithms as well as an overall agreement score. Complete case datasets can be found in the supplemental material Table S4, Table S5 and Table S6. In HGSOC samples, stability of 17 miRNAs were evaluated (Table 5). In these samples the delta-Ct method ranks hsa-miR-23a-3p as most stable and hsa-miR-126-3p as second most stable miRNA. This is in line with Normfinder that ranks these as third and second most stable respectively, with hsa-miR-191-5p as most stable. GeNorm also ranks these two as some of the most stable: with hsa-miR-126-3p and hsa-miR-223-3p as most stable and hsa-miR-23a-3p as the fourth most stable. This is contrasted by BestKeeper that ranks hsa-miR-126-3p as the eighth most stable and hsa-miR-23a-3p as 12th most stable corresponding to the sixth least stable miRNA. Instead, BestKeeper finds hsa-miR-193a-5p as the most stable and hsa-miR-486-5p as second. Interestingly, the four other algorithms find hsa-miR-193a-5p to be the least stable of all miRNAs. Using these different rankings, RefFinder identify hsa-miR-126-3p as most stable and hsa-miR-23a-3p as second most stable followed by hsa-miR-191-5p and hsa-miR-223-3p.

**Table 5:** Stability values and ranking of miRNAs in HGSOC, benign and in all combined samples. Five different algorithms were used on the miRNAs with complete cases in the sample group. RefFinder uses a geometric mean of the rankings from the four others

HGSOC	delta-Ct		BestKeeper		Normfinder		geNorm		RefFinder Rank	
Target	Avg. STDEV.	Rank	MAD	Rank	Stability	Rank	Avg.M	Rank	Geom. mean value	Rank
hsa-miR-126-3p	0.748	2	1.643	8	0.361	3	0.313	1	2.632	1
hsa-miR-23a-3p	0.722	1	1.685	12	0.333	2	0.353	4	3.130	2
hsa-miR-191-5p	0.772	6	1.522	4	0.328	1	0.464	8	3.722	3
hsa-miR-223-3p	0.771	5	1.653	10	0.399	5	0.313	1	3.976	4
hsa-miR-27a-3p	0.751	3	1.763	15	0.408	6	0.336	3	5.335	5
hsa-miR-130a-3p	0.768	4	1.754	14	0.379	4	0.388	5	5.785	6
hsa-miR-21-5p	0.791	7	1.646	9	0.409	7	0.408	6	7.172	7
hsa-miR-24-3p	0.795	8	1.617	6	0.450	8	0.480	9	7.667	8
hsa-miR-193a-5p	1.788	17	0.903	1	1.666	17	0.969	17	8.372	9
hsa-miR-199a-3p	0.953	12	1.558	5	0.682	11	0.583	12	9.434	10
hsa-miR-486-5p	1.683	16	1.106	2	1.549	16	0.860	16	9.514	11
hsa-miR-146b-5p	0.809	9	1.800	16	0.493	9	0.439	7	9.759	12
hsa-miR-101-3p	1.202	15	1.477	3	0.954	15	0.738	15	10.031	13
hsa-miR-221-3p	0.944	11	1.674	11	0.682	11	0.541	11	11.000	14
hsa-miR-143-3p	1.034	13	1.624	7	0.740	13	0.671	14	11.344	15
hsa-miR-103a-3p	0.903	10	1.812	17	0.629	10	0.511	10	11.419	16
hsa-miR-199a-5p	1.045	14	1.734	13	0.818	14	0.628	13	13.491	17
Benign	delta-Ct		BestKeeper		Normfinder		geNorm		RefFinder Rank	
Target	Avg. STDEV.	Rank	MAD	Rank	Stability	Rank	Avg.M	Rank	Geom. mean value	Rank
hsa-miR-191-5p	0.561	1	1.536	2	0.208	1	0.373	4	1.682	1
hsa-miR-27a-3p	0.563	2	1.622	7	0.227	2	0.310	1	2.300	2
hsa-miR-126-3p	0.591	4	1.691	9	0.308	4	0.310	1	3.464	3
hsa-miR-23a-3p	0.565	3	1.753	10	0.268	3	0.356	3	4.054	4
hsa-miR-223-3p	0.664	6	1.549	3	0.407	6	0.437	6	5.045	5
hsa-miR-21-5p	0.680	7	1.569	4	0.454	7	0.468	7	6.086	6
hsa-miR-24-3p	0.624	5	1.801	12	0.403	5	0.392	5	6.223	7
hsa-miR-101-3p	0.919	12	1.358	1	0.781	12	0.631	12	6.447	8
hsa-miR-199a-3p	0.716	9	1.620	6	0.525	8	0.523	9	7.896	9
hsa-miR-103a-3p	0.701	8	1.871	13	0.533	9	0.496	8	9.302	10
hsa-miR-130a-3p	0.767	11	1.647	8	0.573	11	0.574	11	10.158	11
hsa-miR-486-5p	1.149	13	1.591	5	1.058	13	0.711	13	10.238	12
hsa-miR-221-3p	0.738	10	1.791	11	0.570	10	0.544	10	10.241	13
All	delta-Ct		BestKeeper		Normfinder		geNorm		RefFinder Rank	
Target	Avg. STDEV.	Rank	MAD	Rank	Stability	Rank	Avg.M	Rank	Geom. mean value	Rank
hsa-miR-23a-3p	0.611	1	1.766	9	0.272	1	0.372	3	2.280	1
hsa-miR-27a-3p	0.634	2	1.774	10	0.305	3	0.355	1	2.783	2
hsa-miR-126-3p	0.641	3	1.721	7	0.314	4	0.355	1	3.027	3
hsa-miR-191-5p	0.645	4	1.600	3	0.284	2	0.426	5	3.310	4
hsa-miR-223-3p	0.684	6	1.668	5	0.373	5	0.403	4	4.949	5
hsa-miR-24-3p	0.676	5	1.757	8	0.405	6	0.449	6	6.160	6
hsa-miR-21-5p	0.707	7	1.691	6	0.416	7	0.474	7	6.735	7
hsa-miR-486-5p	1.478	13	1.432	1	1.397	13	0.794	13	6.846	8
hsa-miR-101-3p	1.067	12	1.517	2	0.906	12	0.670	12	7.667	9
hsa-miR-199a-3p	0.841	11	1.625	4	0.647	11	0.591	11	8.542	10
hsa-miR-130a-3p	0.737	8	1.791	12	0.459	8	0.504	8	8.853	11
hsa-miR-103a-3p	0.789	9	1.884	13	0.589	9	0.536	9	9.867	12
hsa-miR-221-3p	0.816	10	1.788	11	0.626	10	0.562	10	10.241	13

In benign samples, stability of 13 miRNAs were evaluated (Table 5). Here, hsa-miR-191-5p and hsa-miR-27a-3p is found to be the most and second most stable respectively, by delta-Ct. The results of the Normfinder ranking are, except for a single switch in the order of hsa-miR-199a-3p and hsa-miR-103a-3p, identical to delta-Ct. The most and second most stable miRNAs are therefore also here hsa-miR-191-5p and hsa-miR-27a-3p. GeNorm ranks these in reverse order with hsa-miR-27a-3p and hsa-miR-126-3p as most stable and hsa-miR-191-5p as fourth most stable with hsa-miR-23a-3p as third most stable. BestKeeper ranks hsa-miR-191-5p as second most stable and hsa-miR-27a-3p as seventh most stable with hsa-miR-101-3p. Combining these rankings, RefFinder orders hsa-miR-191-5p, hsa-miR-27a-3p, hsa-miR-126-3p, and hsa-miR-23a-3p as most, second most, third most and fourth most stable respectively.

When combining the two groups (HGSOC and benign), only the miRNAs in common between these groups were evaluated. Opportunely, the 13 miRNAs evaluated for the benign group were all represented in the HGSOC group as well, thus stability of 13 miRNAs were assessed for all samples (Table 5). Here, hsa-miR-23a-3p, hsa-miR-27a-3p, hsa-miR-126-3p and hsa-miR-191-5p ranked as most, second most, third most and fourth most stable respectively. Normfinder mostly agrees on this, however, with a slightly different order with hsa-miR-23a-3p, hsa-miR-191-5p, hsa-miR-27a-3p and hsa-miR-126-3p ordered from most stable to least stable. GeNorm largely agrees with the two others, however it finds hsa-miR-191-5p to be the fifth most stable with hsa-miR-27a-3p and hsa-miR-126-3p as the most stable miRNAs and hsa-miR-23a-3p and hsa-miR-223-3p as the third and fourth most stable, respectively. Interestingly, BestKeeper finds quite different rankings compared to the three other algorithms. Though hsa-miR-191-5p also here is found to be quite stable as the third most stable miRNA, hsa-miR-23a-3p ranks as the ninth most stable, hsa-miR-27a-3p as the 10th most stable and hsa-miR-126-3p as the seventh most stable miRNA. The most stable, second most stable and fourth most stable miRNA are instead found by BestKeeper to be hsa-miR-486-5p, hsa-miR-101-3p and hsa-miR-199a-3p, respectively.

To further validate our findings that hsa-miR-23a-3p and hsa-miR-27a-3p were the most stable miRNAs across HGSOC and benign samples, we performed grouped Normfinder analysis on the combined dataset assessing the most stable pair of miRNAs across the two sample groups. This resulted in hsa-miR-23a-3p and hsa-miR-27a-3p being the most stable pair with a stability value of 0.042 (supplementary Table S7).

## Discussion

Discovery and detection of blood-based miRNA biomarkers largely depends upon the accurate and robust measurement of the presence of miRNAs in an analyzed sample. RT-qPCR is a proven and reliable method for the detection and quantification of miRNA and other nucleotide sequences. However, the accuracy of RT-qPCR quantification is dependent on the selection of a stable reference for normalization [29, 30].

Missing consensus on how to perform quality control and preprocessing of data to identify and assess the stability of reference miRNA candidates also makes direct comparisons challenging. Different algorithms are being used and selection of an algorithm often seems arbitrary [30–33]. Since these algorithms often provide very different recommendations and the outcome of a given study is dependent on the selected references, results could end up appearing random and inconsistent. Moreover, in our experience, the algorithms are quite sensitive to small changes in the datasets, hence the data preprocessing has a major impact on results.

We used the RefSeeker package to perform the RefFinder analysis which provide results from four widely used algorithms (delta-Ct, BestKeeper, Normfinder and geNorm) as well as an overall ranking.

In this study, we found hsa-miR-23a-3p and hsa-miR-126-3p to be the best candidates to use for reference if only HGSOC samples were to be compared (Table 5). For the benign samples hsa-miR-191-5p and hsa-miR-27a-3p were the most stable (Table 5). This indicates that the choice of references and the subsequent results largely depend upon the specific cohort composition and the available reference candidates.

To accommodate this discrepancy between the two sample groups, a combined set was analyzed and hsa-miR-23a-3p and hsa-miR-27a-3p were found to the most stable candidates across both sample types (Table 5). Specific recommendations for good stability value thresholds of most of the algorithms are not clearly communicated and different cut-offs are being used for the different algorithms [30, 34, 35].

For Normfinder a used cut-off stability value of references is 0.5-1. For geNorm 1-1.5 is used. For delta-Ct and BestKeeper, about 1 is common [30, 34, 35]. We found that stabilities for the four miRNAs (hsa-miR-23a-3p, hsa-miR-27a-3p, hsa-miR-126-3p and hsa-miR-191-5p) highlighted in this study all performed below these thresholds (Table 5). Curiously, BestKeeper seem to systematically contradict the three other algorithms. Only one miRNA (hsa-miR-486-5p) in the dataset barely pass a BestKeeper stability threshold of 1.5 (MAD = 1.432) (Table 5).

Interestingly neither hsa-miR-191-5p nor hsa-miR-126-3p was part of the top performing pair in the combined sample group, even though they both performed better in the HGSOC group compared to hsa-miR-27a-3p and better then hsa-miR-23a-3p in the benign group. Nonetheless, both hsa-miR-191-5p and hsa-miR-126-3p were determined to be stable and using these as references could probably also be a viable option.

As previously seen, BestKeeper evaluation of stability is largely in direct opposition to the other algorithms [21]. For example, when looking at stabilities of the HGSOC and benign groups combined, hsa-miR-23a-3p is determined to be the most stable miRNA by delta-Ct and Normfinder, and third most stable by geNorm. However, BestKeeper ranks this miRNA as number 9 out of 13 (Table 5). BestKeeper uses mean absolute deviation of the Cp values for each candidate and in doing so do not consider comparing with other miRNAs taking experimental and technical variations into account. Since it has not yet been determined which algorithms are superior and in which contexts, we implemented RefFinder to provide an overview and compare results. Though this approach has been used in a number of studies it has to our knowledge never been properly validated, emphasizing the need for more precise standardization of methods for miRNA quantifications [28, 36]. It is therefore imperative, that the results from the RefFinder Rank is validated.

In this study we validated the findings using two methods. We used the grouped Normfinder analysis which uses a weighted mean of two parameters inter- and intragroup variations. Adding the intergroup variation into the calculation considers that a given stable miRNA should be stable in both within and between the analyzed sample groups.

To our knowledge, there are no publicly available datasets that contain suitable miRNA RT-qPCR data, to validate our findings in external OC cohorts. Shapira et al. published a study based on miRNA profiles obtained by RT-qPCR on presurgical plasma samples from 42 women with serous EOC, 36 women diagnosed with a benign neoplasm, and 23 comparably age-matched women with no known pelvic mass, however no raw data is provided. Hsa-miR-126-3p was reported downregulated in plasma from 42 serous EOC patients compared with 23 controls [37].

Interestingly, Resnick et al. found hsa-miR-126-3p to be overexpressed in serum from 19 EOC patients compared to that of 11 healthy controls. These discrepancies might be explained by relatively low number of examined patients and differences in the cohort heterogeneity, as exact subtypes of OC being included are not precisely described. Moreover, the normalization strategies are different: Shapira et al. normalized the data by subtracting the mean of complete cases miRNAs expression values in each sample from all miRNAs in that sample, whereas Resnick et al. used two miRNAs (hsa-miR-142-3p and hsa-miR-16) as endogenous stable controls. However, the details on how these normalizers have been chosen were not provided [38].

Hsa-miR-23a-3p has previously been reported to be stable in blood and plasma as well as being unaffected by hemolysis [26]. Indeed, it is used in combination with miR-451, present in high amounts in red blood cells [39], to assess the degree of hemolysis in blood-based samples [26].

Interestingly, U6-snRNA that is often used as a stable endogenous reference for normalization [14, 15] was excluded from the stability analysis in our study. After quality control and filtering U6-snRNA was only represented in 44% HGSOC samples and only 23% of benign tumor samples. Of the 60 HGSOC samples, only 26 U6-snRNA Cp values were below 35 cycles and in the benign samples only 10 out of 48 were below 35. Furthermore, none of these values were below 31 cycles, making the use of U6-snRNA as an endogenous control problematic. It should be noted that in this study RT-qPCR were performed on a limited number of selected targets miRNAs and U6-snRNA.

In this study we found 13 miRNA candidates expressed in all samples. To validate ubiquitous expression of the most stable candidates we compared the results with a previous study performed in our research group (Table S8). In that study, miRNAs were investigated in plasma from two different cohorts each consisting of 95 malignant and 95 benign pelvic mass patients. Here, only hsa-miR-191-5p were present in all samples in both cohorts (n = 365), hsa-miR-126-3p was missing in 1 sample (0.27%), hsa-miR-27a-3p was missing in two samples (0.55%) and hsa-miR-23a-3p was missing in 8 samples (2.19%). Due to the ratio of missing values being very low we assess these to be negligeable.

Additional research needs to be performed to further validate our findings and to explore other suitable reference miRNAs.

As discussed previously, RT-qPCR results are heavily affected by the data composition, robust and trustworthy data require prober data cleaning and quality control. Few papers report their methodology for data evaluation and data point exclusion criteria and if quality control like Spike-in or melt curves are reported, often very little information is provided on the specific use of these [40–42]. Here, we found that using a cut-off for Cp values of 35 cycles, 19.8% (1065) of our dataset Cp values was removed. Additionally, 8.9% (481) of remaining Cp values needed manual evaluation of melt curves and 24.7% of these (119) were excluded from the further analysis. In summary, 22% of the data was excluded in this initial validation. Many uses a Cp cut-off, and 35 cycles is a very common number to use in this respect (Fig. 1). However, at least regarding the LightCycler software, not many describe their approach to handling Cp values that is marked by the software to be of uncertain quality [41].

In this project we used the LightCycler 480 (Roche) in combination with the official LightCycler®480 software for RT-qPCR. This software allowed us to evaluate melt-curves for Cp values that were marked by either the Cp table or the Tm table as of uncertain quality.

It is worth noting that this study is limited by the miRNA targets selected for the RT-qPCR panel used. We are here presenting a workflow for data preprocessing and stability analysis and support and emphasize the notion that these or similar steps must be performed for each individual study [13]. We propose to include a set of candidate references in every RT-qPCR panel and to determine which of these are most stable and ubiquitously expressed in the given sample set. In this respect, the four miRNAs mentioned in here could be included in such a candidate list designed for blood plasma analysis as they in our study are proven stable.

## Conclusion

In this study, we provide the basis for further investigations of stability analysis and exclusion principles when performing miRNA expression studies by RT-qPCR. We found that hsa-miR-126-3p and hsa-miR-23a-3p were most stable among tested miRNAs in HGSOC patients, whereas in the benign samples, hsa-miR-191-5p and hsa-miR-27a-3p were the most stable. When combining the two groups sets, hsa-miR-23a-3p and hsa-miR-27a-3p were found as most suitable endogenous references. The choices were based on the stability rankings provided by RefFinder and discrepancies between various algorithms were observed. U6 detection failed in many of the samples and were found to be unreliable as stable reference. Moreover, to achieve consensus between miRNAs studies across various research groups, access to raw data and more detailed reports regarding raw data handling and pre-processing in terms of inclusion should be provided to enable reliable validation across studies.

### Electronic supplementary material

Below is the link to the electronic supplementary material.

Supplementary Material 1

Supplementary Material 2

Supplementary Material 3

Supplementary Material 4

Supplementary Material 5

Supplementary Material 6

Supplementary Material 7

Supplementary Material 8

## Data Availability

All relevant data are within the paper and its Supporting Information files.
