# Supplementary material for: Identification of stably expressed microRNAs in plasma from high-grade serous ovarian carcinoma and benign tumor patients
Source: Mol Biol Rep. 2023 Nov 7;50(12):10235–47. doi: 10.1007/s11033-023-08795-6 (PMC10676310; doi:10.1007/s11033-023-08795-6)
Supplement: Supplementary file 1 — Supplementary Material 1 [file 11033_2023_8795_MOESM1_ESM.docx]

# Identification of stably expressed microRNAs in high-grade serous ovarian carcinomas and benign ovarian tumors, Molecular Biology Reports (2023).

Patrick HD Petersen^1^, Joanna Lopacinska-Jørgensen^1^, Douglas VNP Oliveira^1^, Claus K Høgdall^2^, Estrid V Høgdall^1*^

*^1^Department of Pathology, Herlev Hospital, University of Copenhagen, 2730 Herlev, Denmark, ^2^Department of Gynecology, The Juliane Marie Centre, Rigshospitalet, University of Copenhagen, 2100 Copenhagen, Denmark.*

Corresponding author:

Prof. Estrid Høgdall

Department of Pathology, Herlev Hospital

University of Copenhagen

Borgmester Ib Juuls Vej 25

2730 Herlev, Denmark

e-mail: [estrid.hoegdall@regionh.dk](mailto:estrid.hoegdall@regionh.dk)

*Table S1: Stable endogenous control and biomarker candidates investigated in the study.*

| **Stable endogenous control candidates** | |
| --- | --- |
| hsa-miR-103a-3p [2], hsa-miR-106b-3p [3], hsa-miR-149-3p [4], hsa-miR-191-5p [2], hsa-miR-24-2-5p[3], hsa-miR-24-3p [3], hsa-miR-302d-3p [3], hsa-miR-92b-5p [3], U6-snRNA | |
|  |  |
| **Biomarker candidates** | **Characteristics** |
| hsa-miR-101-3p | OS^1^ in other than EOC^2^ cancers [5] |
| hsa-miR-1183 | OS [6] |
| hsa-miR-1234 | Resistance to chemotherapy [6] |
| hsa-miR-126-3p | OS [6], miRNA:mRNA signatures associated with OS [7] |
| hsa-miR-1301 | Tumor grade [8], cisplatin resistance [9] |
| hsa-miR-130a | Histologic subtype, advanced FIGO^3^ stage [8] |
| hsa-miR-135a-3p | OS [10], progression‐free survival [10,11] |
| hsa-miR-139-3p | Time to progression [6] |
| hsa-miR-141-3p | OS and progression-free survival [12] |
| hsa-miR-143-3p | OS and progression-free survival in other than EOC cancers [13,14] |
| hsa-miR-146b-5p | OS [15],Tumor grade [8] |
| hsa-miR-193a-5p | Type I or II Tumor [8] |
| hsa-miR-195-5p | Discriminate between benign and malignant cases [16] |
| hsa-miR-199a-3p | Cisplatin-resistance [17] |
| hsa-miR-199a-5p | Cisplatin-resistance [17] |
| hsa-miR-200b-3p | OS and progression-free survival [12] |
| hsa-miR-200c-3p | OS and progression-free survival [12,18], discriminate between benign and malignant cases [16] |
| hsa-miR-205-5p | Recurrence prediction [19], Subtype discrimination [18] |
| hsa-miR-21-5p | Discriminate between benign and malignant cases [16] |
| hsa-miR-221-3p | Discriminate between benign and malignant cases [16] |
| hsa-miR-223-3p | MiRNA:mRNA signatures associated with OS [7] |
| hsa-miR-23a-3p | Progression free survival [6] |
| hsa-miR-23a-5p | MiRNA:mRNA signatures associated with OS [7], progression free survival [6] |
| hsa-miR-27a-3p | OS [20] |
| hsa-miR-27a-5p | MiRNA:mRNA signatures associated with OS [7] |
| hsa-miR-34a | Histologic subtype, Tumor grade, and type I or II Tumor [8] |
| hsa-miR-455-3p | Histologic subtype, type I or II Tumor [8] |
| hsa-miR-486-5p | MiRNA:mRNA signatures associated with OS [7] |
| hsa-miR-506-3p | MiRNA:mRNA signatures associated with OS [7] |
| hsa-miR-595 | Advanced FIGO stage[8] |
| hsa-miR-665 | OS [7] |
| hsa-miR-802 | Time to progression [6], progression free survival [21] |

^1^OS – overall survival in months, ^2^EOC – epithelial ovarian carcinoma, ^3^FIGO – International Federation of Gynecology and Obstetrics staging.

Reprinted from Lopacinska-Jørgensen et al. 2023 [1].

References

[1] Lopacinska-Jørgensen J, Petersen PHD, Oliveira DVNP, Høgdall CK, Høgdall E V. Strategies for data normalization and missing data imputation and consequences for potential diagnostic microRNA biomarkers in epithelial ovarian cancer. PLoS One 2023;18:e0282576. https://doi.org/10.1371/journal.pone.0282576.

[2] Bignotti E, Calza S, Tassi RA, Zanotti L, Bandiera E, Sartori E, et al. Identification of stably expressed reference small non-coding RNAs for microRNA quantification in high-grade serous ovarian carcinoma tissues. J Cell Mol Med 2016;20:2341–8. https://doi.org/10.1111/jcmm.12927.

[3] Lopacinska-Joergensen J, Oliveira DVNP, Hoegdall CK, Hoegdall E V. Identification of Stably Expressed Reference microRNAs in Epithelial Ovarian Cancer. In Vivo 2022;36:1059–66. https://doi.org/10.21873/invivo.12803.

[4] Yokoi A, Matsuzaki J, Yamamoto Y, Yoneoka Y, Takahashi K, Shimizu H, et al. Integrated extracellular microRNA profiling for ovarian cancer screening. Nat Commun 2018;9:2–6. https://doi.org/10.1038/s41467-018-06434-4.

[5] Jiang W, Pan JJ, Deng YH, Liang MR, Yao LH. Down-regulated serum microRNA-101 is associated with aggressive progression and poor prognosis of cervical cancer. J Gynecol Oncol 2017;28:1–10. https://doi.org/10.3802/jgo.2017.28.e75.

[6] Prahm KP, Høgdall C, Karlsen MA, Christensen IJ, Novotny GW, Høgdall E. Identification and validation of potential prognostic and predictive miRNAs of epithelial ovarian cancer. PLoS One 2018;13:e0207319. https://doi.org/10.1371/journal.pone.0207319.

[7] Lopacinska-Jørgensen J, Oliveira DVNP, Novotny GW, Høgdall CK, Høgdall E V. Integrated microRNA and mRNA signatures associated with overall survival in epithelial ovarian cancer. PLoS One 2021;16:1–15. https://doi.org/10.1371/journal.pone.0255142.

[8] Prahm KP, Høgdall CK, Karlsen MA, Christensen IJ, Novotny GW, Høgdall E. MicroRNA characteristics in epithelial ovarian cancer. PLoS One 2021;16:1–18. https://doi.org/10.1371/journal.pone.0252401.

[9] Zhong C, Dong Y, Zhang Q, Yuan C, Duan S. Aberrant Expression of miR-1301 in Human Cancer. Front Oncol 2022;11:1–13. https://doi.org/10.3389/fonc.2021.789626.

[10] Tang W, Jiang Y, Mu X, Xu L, Cheng W, Wang X. MiR-135a functions as a tumor suppressor in epithelial ovarian cancer and regulates HOXA10 expression. Cell Signal 2014;26:1420–6. https://doi.org/10.1016/j.cellsig.2014.03.002.

[11] Fukagawa S, Miyata K, Yotsumoto F, Kiyoshima C, Nam SO, Anan H, et al. MicroRNA-135a-3p as a promising biomarker and nucleic acid therapeutic agent for ovarian cancer. Cancer Sci 2017;108:886–96. https://doi.org/10.1111/cas.13210.

[12] Shi M, Mu Y, Zhang H, Liu M, Wan J, Qin X, et al. MicroRNA-200 and microRNA-30 family as prognostic molecular signatures in ovarian cancer: A meta-analysis. Med (United States) 2018;97:1–9. https://doi.org/10.1097/MD.0000000000011505.

[13] Sharma PC, Gupta A. MicroRNAs: Potential biomarkers for diagnosis and prognosis of different cancers. Transl Cancer Res 2020;9:5798–818. https://doi.org/10.21037/tcr-20-1294.

[14] Tokumaru Y, Asaoka M, Oshi M, Katsuta E, Yan L, Narayanan S, et al. High expression of microRNA-143 is associated with favorable tumor immune microenvironment and better survival in estrogen receptor positive breast cancer. Int J Mol Sci 2020;21:1–17. https://doi.org/10.3390/ijms21093213.

[15] Wilczyński M, Żytko E, Szymańska B, Dzieniecka M, Nowak M, Danielska J, et al. Expression of miR-146a in patients with ovarian cancer and its clinical significance. Oncol Lett 2017;14:3207–14. https://doi.org/10.3892/ol.2017.6477.

[16] Oliveira DNP, Carlsen AL, Heegaard NHH, Prahm KP, Christensen IJ, Høgdall CK, et al. Diagnostic plasma miRNA-profiles for ovarian cancer in patients with pelvic mass. PLoS One 2019;14:1–15. https://doi.org/10.1371/journal.pone.0225249.

[17] Wang Q, Ye B, Wang P, Yao F, Zhang C, Yu G. Overview of microRNA-199a regulation in cancer. Cancer Manag Res 2019;11:10327–35. https://doi.org/10.2147/CMAR.S231971.

[18] Vilming Elgaaen B, Olstad OK, Haug KBF, Brusletto B, Sandvik L, Staff AC, et al. Global miRNA expression analysis of serous and clear cell ovarian carcinomas identifies differentially expressed miRNAs including miR-200c-3p as a prognostic marker. BMC Cancer 2014;14:1–13. https://doi.org/10.1186/1471-2407-14-80.

[19] Sujamol S, Vimina ER, Krishnakumar U. Improving Recurrence Prediction Accuracy of Ovarian Cancer Using Multi-phase Feature Selection Methodology. Appl Artif Intell 2021;35:206–26. https://doi.org/10.1080/08839514.2020.1854988.

[20] LI R, WU H, JIANG H, WANG Q, DOU Z, MA H, et al. FBLN5 is targeted by microRNA-27a-3p and suppresses tumorigenesis and progression in high-grade serous ovarian carcinoma. Oncol Rep 2020;44:2143–51. https://doi.org/10.3892/or.2020.7749.

[21] Ferlay J, Colombet M, Soerjomataram I, Mathers C, Parkin DM, Piñeros M, et al. Estimating the global cancer incidence and mortality in 2018: GLOBOCAN sources and methods. Int J Cancer 2019;144:1941–53. https://doi.org/10.1002/ijc.31937.
