# Supplementary material for: Identification of stably expressed microRNAs in plasma from high-grade serous ovarian carcinoma and benign tumor patients
Source: Mol Biol Rep. 2023 Nov 7;50(12):10235–47. doi: 10.1007/s11033-023-08795-6 (PMC10676310; doi:10.1007/s11033-023-08795-6)
Supplement: Supplementary file 2 — Supplementary Material 2 [file 11033_2023_8795_MOESM2_ESM.docx]

# Identification of stably expressed microRNAs in high-grade serous ovarian carcinomas and benign ovarian tumors, Molecular Biology Reports (2023).

Patrick HD Petersen^1^, Joanna Lopacinska-Jørgensen^1^, Douglas VNP Oliveira^1^, Claus K Høgdall^2^, Estrid V Høgdall^1*^

*^1^Department of Pathology, Herlev Hospital, University of Copenhagen, 2730 Herlev, Denmark, ^2^Department of Gynecology, The Juliane Marie Centre, Rigshospitalet, University of Copenhagen, 2100 Copenhagen, Denmark.*

Corresponding author:

Prof. Estrid Høgdall

Department of Pathology, Herlev Hospital

University of Copenhagen

Borgmester Ib Juuls Vej 25

2730 Herlev, Denmark

e-mail: [estrid.hoegdall@regionh.dk](mailto:estrid.hoegdall@regionh.dk)

Table S7; Normfinder grouped stability, top section of table showing stability of pairs of miRNAs.

Gene1 Gene2 Stability

74 hsa-miR-23a-3p hsa-miR-27a-3p 0.042

49 hsa-miR-191-5p hsa-miR-27a-3p 0.045

32 hsa-miR-126-3p hsa-miR-27a-3p 0.049

39 hsa-miR-130a-3p hsa-miR-23a-3p 0.055

70 hsa-miR-223-3p hsa-miR-24-3p 0.055

34 hsa-miR-130a-3p hsa-miR-191-5p 0.058

44 hsa-miR-191-5p hsa-miR-21-5p 0.059

46 hsa-miR-191-5p hsa-miR-223-3p 0.059

61 hsa-miR-21-5p hsa-miR-24-3p 0.060

24 hsa-miR-126-3p hsa-miR-130a-3p 0.061

60 hsa-miR-21-5p hsa-miR-23a-3p 0.062

69 hsa-miR-223-3p hsa-miR-23a-3p 0.062

64 hsa-miR-221-3p hsa-miR-223-3p 0.067

76 hsa-miR-24-3p hsa-miR-27a-3p 0.070

51 hsa-miR-199a-3p hsa-miR-21-5p 0.071

58 hsa-miR-21-5p hsa-miR-221-3p 0.071

59 hsa-miR-21-5p hsa-miR-223-3p 0.071

53 hsa-miR-199a-3p hsa-miR-223-3p 0.072

48 hsa-miR-191-5p hsa-miR-24-3p 0.074

27 hsa-miR-126-3p hsa-miR-21-5p 0.075

29 hsa-miR-126-3p hsa-miR-223-3p 0.075

56 hsa-miR-199a-3p hsa-miR-27a-3p 0.077

73 hsa-miR-23a-3p hsa-miR-24-3p 0.077

40 hsa-miR-130a-3p hsa-miR-24-3p 0.079

66 hsa-miR-221-3p hsa-miR-24-3p 0.081

71 hsa-miR-223-3p hsa-miR-27a-3p 0.081

22 hsa-miR-103a-3p hsa-miR-27a-3p 0.082

67 hsa-miR-221-3p hsa-miR-27a-3p 0.082

47 hsa-miR-191-5p hsa-miR-23a-3p 0.083

35 hsa-miR-130a-3p hsa-miR-199a-3p 0.085

4 hsa-miR-101-3p hsa-miR-191-5p 0.086

55 hsa-miR-199a-3p hsa-miR-24-3p 0.086

62 hsa-miR-21-5p hsa-miR-27a-3p 0.087

37 hsa-miR-130a-3p hsa-miR-221-3p 0.089

9 hsa-miR-101-3p hsa-miR-23a-3p 0.090

31 hsa-miR-126-3p hsa-miR-24-3p 0.090

38 hsa-miR-130a-3p hsa-miR-223-3p 0.090

45 hsa-miR-191-5p hsa-miR-221-3p 0.090

14 hsa-miR-103a-3p hsa-miR-130a-3p 0.091

10 hsa-miR-101-3p hsa-miR-24-3p 0.092

5 hsa-miR-101-3p hsa-miR-199a-3p 0.093

65 hsa-miR-221-3p hsa-miR-23a-3p 0.093

36 hsa-miR-130a-3p hsa-miR-21-5p 0.095

25 hsa-miR-126-3p hsa-miR-191-5p 0.096

43 hsa-miR-191-5p hsa-miR-199a-3p 0.096

7 hsa-miR-101-3p hsa-miR-221-3p 0.099

52 hsa-miR-199a-3p hsa-miR-221-3p 0.099

54 hsa-miR-199a-3p hsa-miR-23a-3p 0.099

30 hsa-miR-126-3p hsa-miR-23a-3p 0.100

2 hsa-miR-101-3p hsa-miR-126-3p 0.102

8 hsa-miR-101-3p hsa-miR-223-3p 0.104

17 hsa-miR-103a-3p hsa-miR-21-5p 0.106

19 hsa-miR-103a-3p hsa-miR-223-3p 0.106

28 hsa-miR-126-3p hsa-miR-221-3p 0.106
