# Supplementary material for: Identification of stably expressed microRNAs in plasma from high-grade serous ovarian carcinoma and benign tumor patients
Source: Mol Biol Rep. 2023 Nov 7;50(12):10235–47. doi: 10.1007/s11033-023-08795-6 (PMC10676310; doi:10.1007/s11033-023-08795-6)
Supplement: Supplementary file 3 — Supplementary Material 3 [file 11033_2023_8795_MOESM3_ESM.docx]

# Identification of stably expressed microRNAs in high-grade serous ovarian carcinomas and benign ovarian tumors, Molecular Biology Reports (2023).

Patrick HD Petersen^1^, Joanna Lopacinska-Jørgensen^1^, Douglas VNP Oliveira^1^, Claus K Høgdall^2^, Estrid V Høgdall^1*^

*^1^Department of Pathology, Herlev Hospital, University of Copenhagen, 2730 Herlev, Denmark, ^2^Department of Gynecology, The Juliane Marie Centre, Rigshospitalet, University of Copenhagen, 2100 Copenhagen, Denmark.*

Corresponding author:

Prof. Estrid Høgdall

Department of Pathology, Herlev Hospital

University of Copenhagen

Borgmester Ib Juuls Vej 25

2730 Herlev, Denmark

e-mail: [estrid.hoegdall@regionh.dk](mailto:estrid.hoegdall@regionh.dk)

Table S8: Validation cohort. Showing number of samples with missing data for miRNAs in plasma from Malignant and benign ovarian tumor patients.

| Cohort 1 (n=175) | |  |  | Cohort 2 (n=190) | |
| --- | --- | --- | --- | --- | --- |
| miRNA | missing samples |  |  | miRNA | missing samples |
| hsa-miR-106a-5p | 0 |  |  | hsa-miR-106a-5p | 0 |
| hsa-miR-199a-5p | 0 |  |  | hsa-miR-126-3p | 0 |
| hsa-miR-26a-5p | 0 |  |  | hsa-miR-140-3p | 0 |
| hsa-miR-335-5p | 0 |  |  | hsa-miR-142-3p | 0 |
| hsa-miR-451a | 0 |  |  | hsa-miR-145-5p | 0 |
| hsa-miR-484 | 0 |  |  | hsa-miR-152-3p | 0 |
| hsa-miR-142-3p | 0 |  |  | hsa-miR-181b-5p | 0 |
| hsa-miR-191-5p | 0 |  |  | hsa-miR-191-5p | 0 |
| hsa-miR-21-5p | 0 |  |  | hsa-miR-199a/b-3p | 0 |
| hsa-miR-221-3p | 0 |  |  | hsa-miR-20a-5p | 0 |
| hsa-miR-223-3p | 0 |  |  | hsa-miR-214-3p | 0 |
| hsa-mir-92a-3p | 0 |  |  | hsa-miR-21-5p | 0 |
| hsa-miR-93-5p | 0 |  |  | hsa-miR-221-3p | 0 |
| hsa-miR-145-5p | 1 |  |  | hsa-miR-223-3p | 0 |
| hsa-miR-148b-3p | 1 |  |  | hsa-miR-25-3p | 0 |
| hsa-miR-96-5p | 1 |  |  | hsa-miR-26a-5p | 0 |
| hsa-miR-126-3p | 1 |  |  | hsa-miR-27a-3p | 0 |
| hsa-miR-141-3p | 1 |  |  | hsa-miR-335-5p | 0 |
| hsa-miR-27a-3p | 2 |  |  | hsa-miR-372-3p | 0 |
| hsa-miR-346 | 2 |  |  | hsa-miR-378a-3p | 0 |
| hsa-miR-181b-5p | 2 |  |  | hsa-miR-451a | 0 |
| hsa-miR-378a-3p | 2 |  |  | hsa-miR-484 | 0 |
| hsa-miR-20a-5p | 2 |  |  | hsa-mir-92a-3p | 0 |
| hsa-miR-27b-3p | 4 |  |  | hsa-miR-93-5p | 0 |
| hsa-miR-140-5p | 3 |  |  | hsa-miR-96-5p | 0 |
| has-miR-152-3p | 4 |  |  | hsa-miR-148b-3p | 1 |
| hsa-miR-25-3p | 4 |  |  | hsa-miR-373-3p | 1 |
| hsa-miR-23a-3p | 4 |  |  | hsa-miR-29a-3p | 1 |
| hsa-let-7f-5p | 5 |  |  | hsa-miR-27b-3p | 2 |
| hsa-miR-29a-3p | 7 |  |  | hsa-miR-23a-3p | 4 |
| hsa-miR-195-5p | 8 |  |  | hsa-miR-141-3p | 4 |
| hsa-miR-205-5p | 12 |  |  | hsa-miR-205-5p | 6 |
| hsa-miR-214-3p | 12 |  |  | hsa-let-7f-5p | 9 |
| hsa-miR-372-3p | 14 |  |  | hsa-miR-103a-3p | 9 |
| hsa-miR-103a-3p | 21 |  |  | hsa-miR-122-5p | 11 |
| hsa-miR-23b-3p | 38 |  |  | hsa-miR-195-5p | 11 |
| hsa-miR-122-5p | 51 |  |  | hsa-miR-125b-5p | 28 |
| hsa-miR-373-3p | 62 |  |  | hsa-miR-200c-3p | 28 |
| hsa-miR-200c-3p | 63 |  |  | hsa-miR-23b-3p | 42 |
| hsa-miR-125b-5p | 69 |  |  | hsa-miR-200b-3p | 62 |
|  |  |  |  | hsa-miR-346 | 85 |
|  |  |  |  | hsa-miR-182-5p | 94 |
|  |  |  |  | hsa-miR-424-5p |  |
|  |  |  |  | hsa-miR-429 |  |
