# Supplementary material for: Identification of stably expressed microRNAs in plasma from high-grade serous ovarian carcinoma and benign tumor patients
Source: Mol Biol Rep. 2023 Nov 7;50(12):10235–47. doi: 10.1007/s11033-023-08795-6 (PMC10676310; doi:10.1007/s11033-023-08795-6)
Supplement: Supplementary file 5 — Supplementary Material 5 [file 11033_2023_8795_MOESM5_ESM.docx]

# Identification of stably expressed microRNAs in high-grade serous ovarian carcinomas and benign ovarian tumors, Molecular Biology Reports (2023).

Patrick HD Petersen^1^, Joanna Lopacinska-Jørgensen^1^, Douglas VNP Oliveira^1^, Claus K Høgdall^2^, Estrid V Høgdall^1*^

*^1^Department of Pathology, Herlev Hospital, University of Copenhagen, 2730 Herlev, Denmark, ^2^Department of Gynecology, The Juliane Marie Centre, Rigshospitalet, University of Copenhagen, 2100 Copenhagen, Denmark.*

Corresponding author:

Prof. Estrid Høgdall

Department of Pathology, Herlev Hospital

University of Copenhagen

Borgmester Ib Juuls Vej 25

2730 Herlev, Denmark

e-mail: [estrid.hoegdall@regionh.dk](mailto:estrid.hoegdall@regionh.dk)

Table S2: Overview of qPCR samples analyzed by the LightCycler®480 software. Amplifications that were approved by both Cp and Tm calling was approved automatically. Amplifications where a Cp value was not found were automatically marked as absent. The rest of the amplifications were subjected to manual evaluation of their melt curves. Cp > 35 includes both raw Cp values and Cp values adjusted based on the inter plate calibrator.

| Tm\Cp | Approved | Uncertain | Absent | Cp > 35 |
| --- | --- | --- | --- | --- |
| Approved | 3130 | 0 | 90 | 673 |
| Inconclusive | 447 | 4 | 162 | 330 |
| Absent | 14 | 3 | 340 | 39 |
