# Supplementary material for: Identification of stably expressed microRNAs in plasma from high-grade serous ovarian carcinoma and benign tumor patients
Source: Mol Biol Rep. 2023 Nov 7;50(12):10235–47. doi: 10.1007/s11033-023-08795-6 (PMC10676310; doi:10.1007/s11033-023-08795-6)
Supplement: Supplementary file 6 — Supplementary Material 6 [file 11033_2023_8795_MOESM6_ESM.docx]

# Identification of stably expressed microRNAs in high-grade serous ovarian carcinomas and benign ovarian tumors, Molecular Biology Reports (2023).

Patrick HD Petersen^1^, Joanna Lopacinska-Jørgensen^1^, Douglas VNP Oliveira^1^, Claus K Høgdall^2^, Estrid V Høgdall^1*^

*^1^Department of Pathology, Herlev Hospital, University of Copenhagen, 2730 Herlev, Denmark, ^2^Department of Gynecology, The Juliane Marie Centre, Rigshospitalet, University of Copenhagen, 2100 Copenhagen, Denmark.*

Corresponding author:

Prof. Estrid Høgdall

Department of Pathology, Herlev Hospital

University of Copenhagen

Borgmester Ib Juuls Vej 25

2730 Herlev, Denmark

e-mail: [estrid.hoegdall@regionh.dk](mailto:estrid.hoegdall@regionh.dk)

Table S3: Filtering samples based on the UniSp2 and UniSp4 Spike-in controls. Samples were excluded from analysis if its UniSp2 value was an outlier. Samples were also excluded if the difference between UniSp2 and Unisp4 values were either below 5 or above 8. Note that one benign sample were identified both as an outlier with a difference in UniSp2 and UniSp4 below 5.

|  | HGSOC | Benign |
| --- | --- | --- |
| UniSp2 outliers | 0 | 1 |
| Δ_(UniSp2-UniSp4)_ < 5 | 6 | 5 |
| Δ_(UniSp2-UniSp4)_ > 8 | 2 | 0 |
| Total excluded | 8 | 5 |
| Total Remaining | 52 | 43 |
| Note: Some samples are represented twice has both the UniSp2 were an outlier and difference in UniSp2 and UniSp4 are outside desired range. | | |
